# Burden of disease in Lambert-Eaton myasthenic syndrome: taking the patient’s perspective

**DOI:** 10.1007/s00415-024-12206-6

**Published:** 2024-02-29

**Authors:** Sophie Lehnerer, Meret Herdick, Regina Stegherr, Lea Gerischer, Frauke Stascheit, Maike Stein, Philipp Mergenthaler, Sarah Hoffmann, Andreas Meisel

**Affiliations:** 1 Department of Neurology with Experimental Neurology, Charité – Universitätsmedizin Berlin, Corporate Member of Freie Universität Berlin and Humboldt-Universität Zu Berlin, Charitéplatz 1, 10117 Berlin, Germany; 2 Department of Neurology With Experimental Neurology, Charité – Universitätsmedizin Berlin, corporate member of Freie Universität Berlin and Humboldt-Universität Zu Berlin, Neuroscience Clinical Research Center, Charitéplatz 1, 10117 Berlin, Germany; 3 Center for Stroke Research Berlin, Charité – Universitätsmedizin Berlin, Charitéplatz 1, 10117 Berlin, Germany; 4 Berlin Institute of Health at Charité – Universitätsmedizin Berlin, Digital Health Center, Charitéplatz 1, 10117 Berlin, Germany; 5 Institute of Biometry and Clinical Epidemiology, Charité – Universitätsmedizin Berlin, corporate member of Freie Universität Berlin and Humboldt-Universität Zu Berlin, Charitéplatz 1, 10117 Berlin, Germany; 6 Radcliffe Department of Medicine, University of Oxford, Oxford, UK

**Keywords:** Lambert-Eaton myasthenic syndrome, Quality of life, Burden of disease, Real-world setting

## Abstract

**Background:**

Lambert-Eaton myasthenic syndrome (LEMS) is an autoimmune-mediated neuromuscular disorder leading to muscle weakness, autonomic dysregulation and hyporeflexia. Psychosocial well-being is affected. Previously, we assessed burden of disease for Myasthenia gravis (MG). Here, we aim to elucidate burden of disease by comparing health-related quality of life (HRQoL) of patients with LEMS to the general population (genP) as well as MG patients.

**Methods:**

A questionnaire-based survey included sociodemographic and clinical data along with standardized questionnaires, e.g. the Short Form Health (SF-36). HRQoL was evaluated through matched-pairs analyses. Participants from a general health survey served as control group.

**Results:**

46 LEMS patients matched by age and gender were compared to 92 controls from the genP and a matched cohort of 92 MG patients. LEMS participants showed lower levels of physical functioning (SF-36 mean 34.2 SD 28.6) compared to genP (mean 78.6 SD 21.1) and MG patients (mean 61.3 SD 31.8). LEMS patients showed lower mental health sub-scores compared to genP (SF-36 mean 62.7 SD 20.2, vs. 75.7 SD 15.1) and MG patients (SF-36 mean 62.7 SD 20.2, vs. 66.0 SD 18.). Depression, anxiety and fatigue were prevalent. Female gender, low income, lower activities of daily living, symptoms of depression, anxiety and fatigue were associated with a lower HRQoL in LEMS.

**Discussion:**

HRQoL is lower in patients with LEMS compared to genP and MG in a matched pair-analysis. The burden of LEMS includes economic and social aspects as well as emotional well-being.

**Trial Registration Information:**

drks.de: DRKS00024527, submitted: February 02, 2021, https://drks.de/search/en/trial/DRKS00024527.

**Supplementary Information:**

The online version contains supplementary material available at 10.1007/s00415-024-12206-6.

## Background

Lambert-Eaton myasthenic syndrome (LEMS) is a rare neuromuscular autoimmune disease. Prevalence based on a Dutch and an American cohort study is estimated around 3.3–3.4 per million inhabitants [1, 35]. Specific epidemiologic data for Germany is missing. The most common age of onset ranges from 55–60 years [1, 42]. Antibodies targeted against the presynaptic voltage-gated calcium channels (VGCC) are detected in about 90% of patients [17, 26]. Symptoms include muscle fatigability and proximally pronounced weakness, hypo- or areflexia and autonomous dysregulation [29]. Epidemiological studies estimate LEMS to be associated to carcinoma in up to 60% of cases [36]. For paraneoplastic LEMS (pLEMS) small cell lung cancer (SCLC) is the leading tumour entity, however further tumours have been described to be associated with LEMS including Merkel cell carcinoma, neuroendocrine tumours, prostate cancer and lymphomas [24, 40]. Therapeutic options for LEMS encompass 3,4-diaminopyridine (3,4-DAP) or 3,4-diaminopyridine phosphate (3,4-DAPP) as first-line options [24] as well as acetylcholinesterase inhibitors and immunosuppressive agents also commonly used in treatment of myasthenia gravis (MG) [35]. Additionally, for patients with pLEMS, tumour therapy needs to be addressed. Median survival of pLEMS patients with SCLC is longer than for SCLC patients without LEMS [18]. While LEMS symptoms typically present prior to clinical symptoms of SCLC, allowing for early tumour screening, there is evidence, that improved survival in SCLC patients with pLEMS may not solely be dependent on early diagnostic measures but that there may also be biochemical or immunologic causes [20]. Life expectancy of LEMS patients without neoplasia is comparable to the general population [18]. Most LEMS patients reach maximum disease severity before or within the first year of diagnosis [18]. Functional impairment typically improves within 1 year after diagnosis for both autoimmune LEMS (aiLEMS) and pLEMS patients, though pLEMS patients report higher levels of functional impairment throughout the disease course [18].

Few studies have analysed health-related quality of life (HRQoL) and perceived physical and mental health in LEMS patients. HRQoL has been indicated to be reduced in LEMS based on assessments of the Short Form-36 (SF-36) [18] as well as the EQ-5D (European Quality of Life 5 Dimensions) questionnaire [6]. Correlating with higher prevalence, more data are available on HRQoL in autoimmune MG. MG is a neuromuscular disease characterized by antibodies against the acetylcholine receptor protein complex-mediating blockage of postsynaptic receptors and partly overlapping symptoms (e.g. muscle fatigability) with LEMS. We and others have found that HRQoL is lower for MG patients compared to general population (genP) and that there are patient characteristics, such as depression and anxiety, associated with worse HRQoL [2, 16, 38]. However, for aiLEMS and pLEMS, data on many aspects affecting the overall burden of disease and thereby loss of health are sparse or missing.

We aim to elucidate the burden of disease in LEMS as well as associated risk factors. Accordingly, questionnaire-based data from LEMS patients were analysed and compared to the general population and MG patients.

## Methods

### Data collection

In February 2021, the members of the German Myasthenia Gravis Society (Deutsche Myasthenie Gesellschaft, DMG) with LEMS received via mail the study information and the questionnaire as well as a pre-stamped envelope addressed to the coordinating study centre. The study participants (SP) were instructed to return their completed questionnaire without any further identifying information to ensure the anonymity of the survey. No refund was given. Returned questionnaires were accepted within the cut-off date of 31st May 2021.

### Questionnaire

The questionnaire included demographic data (gender: female/male/diverse, age, marital status/partnership, family planning), educational status, employment, income, fear of old age poverty and possession of a severely disabled person card (in Germany delivered at a certain degree of disability ranging from 10 (mild) to 100 (very severe)). Educational status was graded into three groups (low, medium, high) based on information on the highest level of education according to the CASMIN classification [15]. Information of net household-income was based on income categories: "Less than 1000€", "Between 1000€ and 2499€", "Between 2500 and 5000€" and "More than 5000€".

Clinical data included age at symptom onset, age at medical diagnosis, current symptoms, symptom severity (low, medium, high), antibody status (acetylcholine receptor antibody (Ach-R-Antibodies), Voltage-Gated Calcium Cannel antibody of *P*/*Q* Type (VGCC-Antibodies), seronegative (no antibody detection)), comorbidities including other autoimmune diseases, tumours including chronological context, current LEMS-specific medication (cholinesterase inhibitors (i.e. pyridostigmine (sustained release)), potassium channel blockers (i.e. 3,4-DAP or 3,4-DAPP), glucocorticosteroids, long-term immunosuppressants (azathioprine, mycophenolate mofetil, methotrexate, cyclosporine A), monoclonal antibodies (rituximab), plasmapheresis (PE)/immunoadsorption (IA), intravenous immunoglobins (IVIG) including dosage/frequency, co-medication (antidepressants, painkillers), side-effects and treatment satisfaction.

Most questions were asked with a checkbox option, always specified to be answered as a single or multiple-choice option. Only few questions were asked as free-text format. The questionnaires were scanned and processed with the software TeleForm (OpenText), version 10.9.1.

### Standardized scores

To further assess the burden of disease standardized scores in German language were integrated in the questionnaire (SF-36 (Short Form Health, i.e. general HRQoL) [25, 39], CFQ11 (Chalder Fatigue scale) [5, 9, 23], ESSI-D (ENRICHED Social Support Inventory) [8, 13] and HADS (Hospital anxiety and depression scale) that encompasses a subscale of anxiety (HADS-A) and depression (HADS-D) [3, 7, 45]). In the absence of questionnaires specifically designed and validated for LEMS, we used questionnaires tailored to MG-specific symptoms as there is an overlap between MG and LEMS, i.e. MG-QoL15 (Myasthenia gravis quality of life, i.e. MG-specific HRQoL) [4] and MG-ADL (Myasthenia gravis activities of daily living profile) [43]. In the SF-36 (0–100-point scale) and the ESSI-D (5–25-point scale), the higher the score, the better is the patients´ condition. Whereas in the MG-QoL15 (0–60-point scale), the MG-ADL (0–24-point scale), the HADS (0–21-point scale for HADS-A and HADS-D) and the CFQ11 (0–33-point scale) a high score indicates a worse condition. Additional to the Likert format, the CFQ11 offers a binary scoring where 4 points or more equate severe fatigue [5]. In the ESSI-D low social support is defined as a sum score of 18 or less and at least two items with 3 or less points [8]. With a HADS sub-score scoring 8 points or more, participants are defined as having substantial grades of anxiety or depression [3].

### Statistical analysis

The statistical calculations were performed using IBM SPSS Statistics for Windows, Version 27.0. Armonk, NY: IBM Corp. and R (version 4.2.2) [31] software. Appropriate descriptive statistics (mean, standard deviation, median, interquartile range, absolute and relative frequencies) are presented depending on the scale and distribution of the variables. To test for group differences, parametric and non-parametric measures were used. A two-sided significance level of *α* = 0.05 was used. No adjustment for multiple testing was applied in this exploratory study. Linear mixed regression models adjusted for gender, age, educational status, income, and partnership status were calculated (random intercept models, random intercept for matching ID) for the analyses of the differences between LEMS patients, MG patients, and controls in the SF-36 subdomains *physical functioning* and *emotional well-being*. Furthermore, interactions between disease status (LEMS/MG/Control) and age or sex were included. The multivariable analysis was carried out in the full analysis set including estimated values in case of missing values. Multiple imputation (*m* = 20 datasets) was used to estimate missing values by using predictive mean matching and chained equations. Twenty complete datasets were created and separately analysed. The results were then combined using Rubin’s rules [32].

Imputation of missing values using the SF-36:

To calculate the subscale scores of the SF-36, following the instructions of Morfeld et al. [25], missing values were replaced by the mean values of the existing items of the same subscales, if at least 50% of the items were answered. For number of missing values with and without imputation of all subscales, see supplement 1.

### Matched controls

To compare HRQoL to the general population (controls), data from a German-wide representative study were used [12] (German Health Interview and Examination Survey for Adults, DEGS1, 2008–2011). The Robert Koch Institute conducted this study and aimed to repeatedly collect representative data on the health status, health-related behaviour, healthcare and living conditions of adults over the age of 18 residing in Germany. To compare the HRQoL to a MG population, the data from Lehnerer et al. 2022 [16] were used. That data were collected with a similar questionnaire as used for the LEMS patients in this study. These two populations were matched to the LEMS patients using exact matching by gender and age groups (25–49), (50–59), (60–69), (70 + years) in a ratio of 1:2.

### Net diagrams

In order to present various aspects of the burden of disease holistically in net diagrams, the different score values of MG-ADL, MG-QoL15, HADS, ESSI-D, CFQ11 and SF-36 subdomains were levelled on a unidirectional scale from zero (no complaints) to 100 points (strongest restrictions).

### Data availability

Data not provided in the article because of space limitations may be shared (anonymized) at the request of any qualified investigator for purposes of replicating procedures and results.

## Results

### Response analysis

Of the 74 contacted members of the DMG with known LEMS diagnosis 47 sent back the questionnaire. No SP had to be excluded retrospectively from analysis as all SP met the inclusion criteria i.e., age ≥ 18 years, self-reported diagnosis of LEMS, no diagnosis of MG. The overall response rate was 63.5%.

### Patient characteristics

**Fig. 1:**
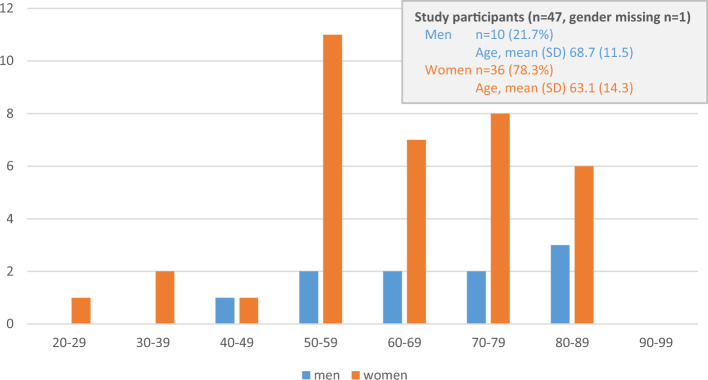
Age distribution of study participants (SP), men (blue) and women (orange), 1 missing (gender). The gender ratio and age distribution of SP did not differ significantly from the entire group of German Myasthenia Gravis Association (DMG) members (*n* = 74, response rate 64.5%)

Mean age of SP was 64.3 years (SD 13.7). The age distribution considering gender is shown in Fig. 1, with 10 men (21.7%) and 36 women (78.3%) (1 missing in gender) participating at the survey. The gender ratio and age distribution of SP did not differ significantly from the entire group of contacted DMG-members.

Mean age of symptom onset was 48.7 (SD 14.0) years, with earlier start of symptoms in women (41.8, SD 19.6) than in men (52.8, SD 12.9). Latency from symptom onset to diagnosis was a mean of 2.8 years in men (SD 4.1) and 4.2 years (SD 9.0) in women. The mean disease duration since diagnosis was 11.8 (7.7) years (Supplement 2). Age at diagnosis did not differ significantly between men and women or pLEMS and aiLEMS, respectively (Table 1).

**Table 1:** Overview: Age at diagnosis, MG-ADL, MG-QoL15, HADS, CFQ, ESSI-D in subgroups (men vs. women, pLEMS vs. aiLEMS, high symptom severity vs. low/medium symptom severity)

Parameter	Missing per row (n)	All (*n* = 47)	Men (*n* = 10, 1 missing)	Women (*n* = 36, 1 missing)	pLEMS (*n* = 10, 0 missing)	aiLEMS (*n* = 37, 0 missing)	high symptom severity (*n* = 6, 0 missing)	low/medium symptom severity (*n* = 41, 0 missing)
Age at diagnosis Median (IQR)	1	51 (48;60)	50 (47;61)	51 (50;59)	50 (49;56)	51 (48;60)	52 (51;55)	51 (47;60)
*p*-values		-	0.917		0.581		0.650	
MG-ADL Median (IQR)	1	6 (3;9)	4.5 (2.3;6.8)	6 (4;9)	7 (3;9)	5.5 (3.8;8.3)	6 (5;9.3)	6 (3;9)
*p*-values		–	0.251		0.788		0.537	
MG-QoL15 Median (IQR)	15	24.5 (16;30)	15 (11.8;16.8)	25 (19;30)	25 (17.5;28.5)	22 (16;30)	43 (38;44)	21 (15;28.5)
*p*-values		–	0.065		0.973		0.002	
HADS Median (IQR)	4	12 (6;17)	9.5 (3.3;15.5)	11.5 (8;17)	10 (8;19)	12 (6;17)	15.5 (12.8;17.5)	11 (5.5;17)
*p*-values		–	0.282		0.877		0.162	
HADS-A ≥ 8 p, n (%)	3	16 (36.4)	3 (30)	12 (35.3)	5 (50)	11 (33.3)	3 (50)	13 (34.2)
HADS-D ≥ 8 p, n (%)	2	14 (31.1)	3 (30)	10 (29.4)	2 (22.2)	12 (33.3)	3 (50)	11 (28.2)
CFQ sum (Likert) Median (IQR)	9	22.5 (19;24.8)	21 (17.5;24)	22 (20;26)	21.5 (18.8;24.5)	22.5 (19.5;24.8)	23 (22;24)	22 (19;25)
*p*-values		–	0.236		0.473		0.809	
CFQ ≥ 4 (Binary) n (%)		38 (100)	8 (100)	29 (100)	8 (100)	30 (100)	5 (100)	33 (100)
ESSI-D ≤ 18 p, *n* (%)	3	10 (22.7)	1 (10)	8 (24.2)	0 (0)	10 (29.4)	1 (16.7)	9 (23.7)
*p*-values		–	0.332		0.051		0.703	

*P* values indicate significance level of Chi-Square-Tests (ESSI-D) or Mann–Whitney-*U*-Test (all other) between men and women, or pLEMS and aiLEMS or high symptom severity and low/medium symptom severity

*pLEMS* paraneoplastic Lambert-Eaton myasthenic Syndrome, *aiLEMS* autoimmune LEMS, *MG-QoL15* Myasthenia gravis quality of life, *MG-ADL* Myasthenia gravis activities of daily living profile, *CFQ-11* Chalder Fatigue scale, *ESSI-D* ENRICHED Social Support Inventory, *HADS* Hospital anxiety and depression scale, *HADS-A* anxiety subscale of HADS, *HADS-D*, depression subscale of HADS, p points

Overall, severity of LEMS was rated as moderate by most (70.2%) SP (Table 2), with a difference in severity between men and women: Only women reported a high disease severity (*n* = 6 of 36 women, *n* = 0 of 10 men). Median MG-ADL sum score was 6 (IQR 3;9). Symptoms were counted as present if responses to MG-ADL sub items other than “normal” were selected by the SP. Beside symptoms registered in the MG-ADL manifestations of autonomic dysfunction were highly prevalent and the majority of SP reported mouth dryness (80.9%) as well as dry eyes (74.5%). Of the 10 male SP six SP reported erectile dysfunction (60%). More than half (56.5%) of the SP reported a positive antibody status. Most commonly, VGCC-Antibodies were reported by 23 of 26 SP (88.5%). 3 of 26 (11.5%) SP reported isolated AchR-Antibodies and 4 of 26 (15.4%) SP reported both VGCC-Antibodies and AchR-Antibodies. Seronegativity was reported by 14.6% of SP and 28.3% of SP reported not to know if an antibody had been detected. About one-fifth (21.3%) of SP reported a history of malignant neoplasia and were defined as pLEMS. Apart from bronchial carcinoma (2/10), reported tumours were: Thyroid cancer, bowel cancer, melanoma, basalioma (2), pancreas carcinoma and suspected ovarian carcinoma. The time of diagnosis of the neoplasia was in 3 SP *before* and in 5 SP (2 missing values) *after* first signs of muscle weakness. At least one comorbid disease was reported by 70.2% of all SP with cardiovascular diseases most common (42.6%) followed by other autoimmune diseases (40%) (Supplement 2).

**Table 2:** Clinical characteristics of study participants (Ach-R-Abs = Acetylcholine receptor antibodies, VGCC-Abs = Voltage-gated calcium channel antibodies of *P*/*Q* Type)

Disease severity (missing *n* = 0)		*n*	%
**Low**		**8**	**17.0**
Women		6	16.7
Men		2	20.0
**Moderate**		**33**	**70.2**
Women		24	66.7
Men		8	80.0
**High**		**6**	**12.8**
Women		6	16.7
Men		0	0.0

Symptoms (≥ 1 point/item in the MG-ADL)	Missing	*n*	%
Fatigue of the eyelids	1	34	73.9
Difficulty to breathe	0	32	68.1
Double vision	0	23	48.9
Difficulty in getting up from the chair	0	39	83.0
Difficulty to chew	0	19	40.4
Difficulty to talk	0	15	31.9
Difficulty to swallow	0	15	31.9
Difficulty in brushing teeth (upper limb strength)	0	22	46.8
**MG-ADL sum score (median, IQR)**	**1**	6	**3/9**

Accompanying symptoms (missing *n* = 0) (within last 4 weeks, multiple answers possible)		*n*	%
Mouth dryness		38	80.9
Sicca symptoms (dry eyes)		35	74.5
Headache		26	55.3
Cognitive Impairment		26	55.3
Dizziness		23	48.9
Bladder voiding disorder		21	44.7
Blood pressure fluctuation		18	38.3
Obstipation		13	27.7
Reduced perspiration		9	19.1
Erectile dysfunction (/10 men)		6	60

Antibody status (missing *n* = 1)		*n*	%
Antibodies detected (multiple answers possible)		26	56.5
VGCC-Abs (total)		23	88.5
Ach-R-Abs (total)		7	26.9
No antibodies (= seronegative)		7	14.6
“I do not know”		13	28.3

Tumor (1 tumor specification missing)		*n*	%
Yes		10	21.3
Bronchial carcinoma		2	20
Thymus carcinoma		0	0
Prostate carcinoma		0	0
Lymphoma		0	0
Other tumor*		7	70

Time of diagnosis of the tumor (missing *n* = 2)		*n*	%
Before first signs of muscle weakness		3	37.5
After first signs of muscle weakness		5	62.5
“I do not know”		0	0

Actual status (medication) (missing *n* = 1)		*n*	%
No symptoms and no medication > 1 year		1	2.2
No symptoms under medication		2	4.3
With medication improvement of symptoms		31	67.4
Despite medication no change in symptoms		7	15.2
Despite medication worse symptoms		4	8.7
Despite medication myasthenic crisis		1	2.2

Therapy satisfaction	Missing/not applicable	*n*	%
Satisfied with medication	1	36	78.3
Side effects under medication	1	25	54.3
Stop of medication in the past	11	18	50.0
…because of side effects	11	9	50.0
…because of alternating blood test results	11	11	61.1
…because of lack of efficacy	11	2	11.1
“I do not know”	11	1	5.6

*Thyroid cancer, bowel cancer, melanoma, basalioma (2), pancreas carcinoma and suspected ovarian carcinoma mentioned in free text specification

First-line symptomatic therapy with 3,4-DAP was used by 37.8% of SP and 3,4-DAPP by 48.9% of SP. Symptomatic treatment with pyridostigmine or pyridostigmine sustained release used 40% and 24.4% of all SP, respectively. Steroids were used by 24.4% of SP. Among the steroid-sparing immunosuppressants azathioprine was most common (44.4%) followed by rituximab (11.1%). Reported measures for treatment escalation were IVIG (46.7%), and plasmapheresis or immunoadsorption (6.7%) (Supplement 3).

Painkillers were used regularly by 19.1% of all SP and 13.3% took antidepressants (Supplement 2). Asking for therapy response, 2.2% of SP reported no intake of medication and no symptoms for more than 1 year (so-called *complete stable remission*). Less than 5% of SP (4.3%) reported *pharmacologic remission* (no symptoms under medication), whereas 67.4% stated *minimal manifestations* (symptoms under medication, although medication improves symptoms). 15.2% reported to have *unchanged status* (i.e. no change in symptoms under medication) and 8.7% reported *worse status*. Overall, 78.3% of SP are satisfied with their current medication (Table 2). Of all SP 54.3% stated to experience current side effects under medication; 36.6% reported stop of medication due to side effects (50%) or due to abnormal laboratory findings (61.1%) or due to lack of efficacy (11.1%) (Table 2, multiple answers possible).

Of all SP, 70.2% were living in a partnership (Table 3). In the subgroup of SP, who were separated or divorced (*n* = 6), LEMS played no role as reason for separation. LEMS has influenced the family planning of 22.2% patients (Table 3). Female patients who had LEMS onset before or during the period of family planning, 100% stated that LEMS affected family planning. Before having experienced first symptoms of LEMS, more than half of the SP (55.3%) were in full-time employment and 23.4% in part-time employment (Table 3). Formerly working patients were asked if they had experienced limitations regarding employment due to LEMS; this was affirmed by 71.4% of SP (Table 3). Most of the SP had a disabled person's card (91.3%) with a median degree of disability of 70 (IQR 50;80), consistent with a moderate degree of disability.

**Table 3:** Sociodemographic characteristics of study participants

Marital status (missing *n* = 0)	*n*	%
Married, living together with the partner	30	63.8
Married, living separate from the partner*	1	2.1
Single*	4	8.5
Widowed*	7	14.9
Divorced*	5	10.6
* Of which living in partnership (missing *n* = 0)	3	17.6
Living in partnership (married or not married)	33	70.2

LEMS was cause of separation (in case of separation or divorce)	*n*	%
LEMS was no cause of separation	6	100.0
LEMS was of minor importance	0	0.0
LEMS was of medium importance	0	0.0
LEMS was of high importance	0	0.0

LEMS affecting family planning (missing *n* = 14)	*n*	%
Yes	**6**	**22.2**
Male	0	0
Female	6	18.8
Male before or during family planning	0	0
Female before or during family planning (missing n = 1)	6	100

Employment level before LEMS symptoms (missing *n* = 1)	*n*	%
Full-time employment	25	54.3
Part-time employment	11	23.9
Pensioner, retiree or in early retirement	7	15.2
Not gainfully employed	3	6.5

Limitations regarding employment because of LEMS? (referring to 39 working patients, including 9 missing)	*n*	%
Yes	25	83.3
Kind of limitations (multiple answers possible)		
…reducing working hours	9	36.0
…recurrent occupational disability	7	28.0
…unemployment	0	0.0
…professional disability	3	12.0
…incapacity to work	12	48.0
…multiple answers selected	5	20.0

Severely disabled person card (missing n = 1)	*n*	%
No	4	8.7
No, but request made	0	0.0
Yes	42	91.3
	Median	IQR
Degree of disability	70	50/80

Net household income (unweighted) (missing *n* = 10)	*n*	%
< 1000€	0	0.0
1000€—2499€	13	35.1
2500€—5000€	16	43.2
> 5000€	8	21.6

Being afraid of poverty in old age (missing *n* = 10)	*n*	%
Yes	11	23.4
…this is due to LEMS (missing *n* = 1)	10	100

Four categories are marked with symbol (asterisk): Married, living separate from the partner, single, widowed, divorced. Line "Of which living in a partnership" referes to thises four categories. To indicate this, the asterisk is used

The majority of SP (43.2%) had an unweighted net household income between EUR 2,500 and EUR 5,000 per month (further details Table 3). Being afraid of old age poverty was affirmed by 11 (23.4%) respondents, among them 10 SP traced this fear back to LEMS (1 missing).

### Lower HRQoL (SF-36) of LEMS patients: a matched-pair comparison with the German general population (genP) and patients with myasthenia gravis

**Fig. 2:**
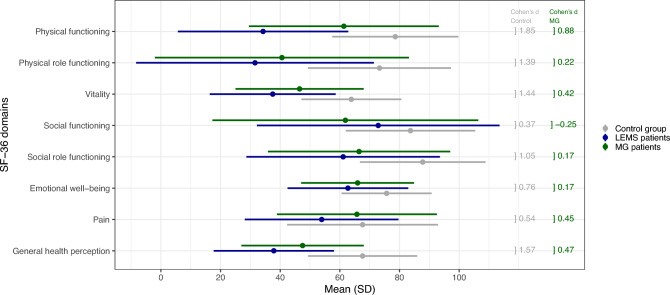
SF-36 score comparison in subdomains: Mean values (and standard deviation, SD) of LEMS patients, *n *= 46 (blue), control group (general population), *n* = 92 (grey) and MG patients (green) (MG patients are *n* = 92 matched controls from the MyaBoD Study [16]. A Cohen’s *d* >0.5 indicates a high effect, 0.3–0.5 medium effect, 0.1–0.3 low effect and < 0.1 no effect

The education level of our patient population was higher compared to the control group (Supplement 4). More SP (63.9%) were in the high-income group compared to the control group (16.3%). While more participants of the control group were in the medium- (34.8% vs. 16.7%) and low-income group (48.9% vs. 19.4%). Figure 2 presents mean values of each of the eight domains of the SF-36. All mean values of LEMS-patients were lower compared to the control group with high statistical effect for the domains *Physical functioning*, *Physical role functioning*, *Vitality* and *Social role functioning* as well as *emotional well-being*, *Pain* and *General health perception*. In a second step, we compared HRQoL by matched-pair comparison for LEMS and MG using data from a previous publication (Supplement 4) [16]. There were more LEMS SP (63.9%) in the high-income group compared to the MG group (46.1%). More MG patients were in the medium (23.1% vs.16.1%) as well as low (30.8% vs. 19.4%) income groups. As represented in Fig. 2 all mean values of LEMS SP apart from *Social functioning* were lower compared to MG patients. Mean values of LEMS-patients were lower compared to the MG group with high statistical effect for *Physical functioning*.

### Worse physical functioning (SF-36) in LEMS patients compared to general population and patients with myasthenia gravis

In multivariable analyses, LEMS patients were 49 (95% CI 33–65) and 31 (95% CI 16–46) points lower in *physical functioning* compared to genP and MG-patients, respectively (linear mixed regression models adjusted for gender, age, educational status, income and partnership status, Table 4). Difference between genP and LEMS and, respectively, MG- and LEMS-patients varied by age group. In both group comparisons, women reported lower values of *physical functioning* than men did.

**Table 4:**
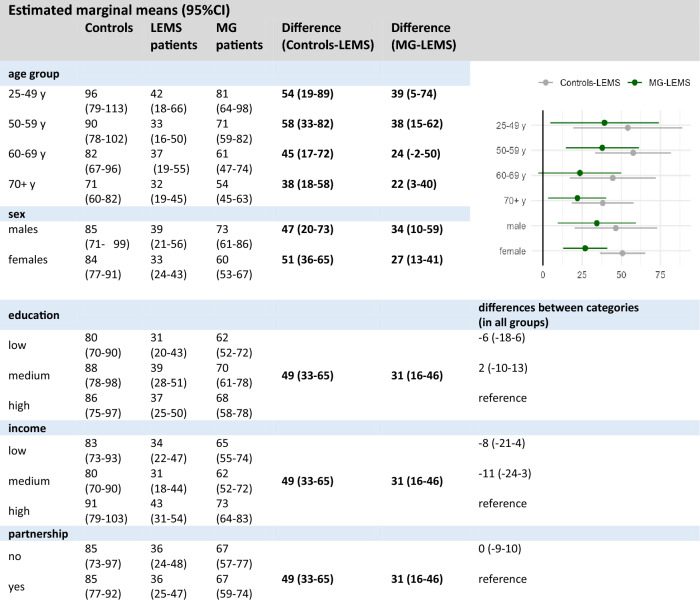
Multivariable analysis on physical functioning (SF-36) (combined results after multiple imputation, *n* = 230). (marginal means and 95%CI, model included interaction effect for group*sex and group*age group)

Further similar associations of income and education with physical functioning were present in all groups: Low and medium income, as well as low education were associated with lower levels of physical functioning compared to the particular reference group.

### Worse emotional well-being (SF-36) in LEMS patients compared to general population and patients with myasthenia gravis

In multivariable analyses of the SF-36 domain *emotional well-being*, LEMS-patients reported lower values than genP (mean difference 9 points, 95% CI − 1–20). However, the differences were relatively small (Supplement 5). A pronounced difference was found for genP and LEMS in the highest age group 70 + (20, 95% CI 7–33). Women reported slightly lower values of emotional well-being than men in the comparison of LEMS and genP. In comparison to MG-patients, LEMS-patients mean values in multivariable analyses were lower (mean difference 4 points, 95% CI − 6–14) (Supplement 5). Differences were found across age groups, though in the group of 50–59 years MG-patients even had slightly lower mean values than LEMS SP (-2, 95% CI − 18–13).

The emotional well-being of highly educated SP was higher compared to those with lower education levels. There was no pronounced difference in emotional well-being by income and partnership status between all groups.

### Overall burden of disease

SP with high symptom severity showed significantly lower quality of life compared to SP with low or medium symptom severity. Compared to men, median scoring of women with LEMS indicated higher levels of difficulties in activities of daily living (MG-ADL), lower quality of life represented by higher MG-QoL15-scores, more symptoms of anxiety and depression (HADS), and of fatigue (CFQ11) as well as lower perceived social support (ESSI-D). However, none of these differences were statistically significant (Table 1). Interestingly, low social support, defined as less than 18 points in the ESSI-D, was reported by almost one third of aiLEMS SP (29.4%) vs. 0% in the pLEMS SP (*p* = 0.051).

**Fig. 3:**
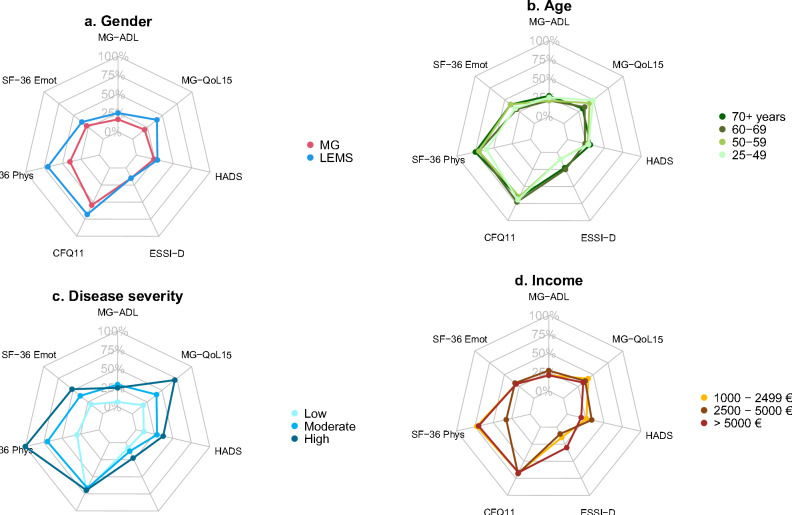
Net diagrams integrating the medians of the Myasthenia gravis Activities of Daily Living Score (MG-ADL), the Myasthenia gravis Quality of life Score (MG-QoL15), the Hospital Anxiety and Depression Scale (HADS), the ENRICHD Social Support Inventory (ESSI-D), the Chalder Fatigue Scale (CFQ11) and the Physical Functioning (SF-36 Phys) and Emotional wellbeing (SF-36 Emot) domain of the Short Form 36 (SF-36) in different subgroups: **a** Gender, **b** age groups, **c** groups of different disease severity and **d** net household income groups. The different score were levelled on a unidirectional scale from zero (no complaints) to 100 points (strongest restrictions), i.e.the further out the lines are in the net, the higher and worse the single score value: Women (**a**), old patients (**b**), patients with high disease severity (**c**) and low income (**d**) do have the highest burden of disease, composed of high single score values

**Fig. 4:**
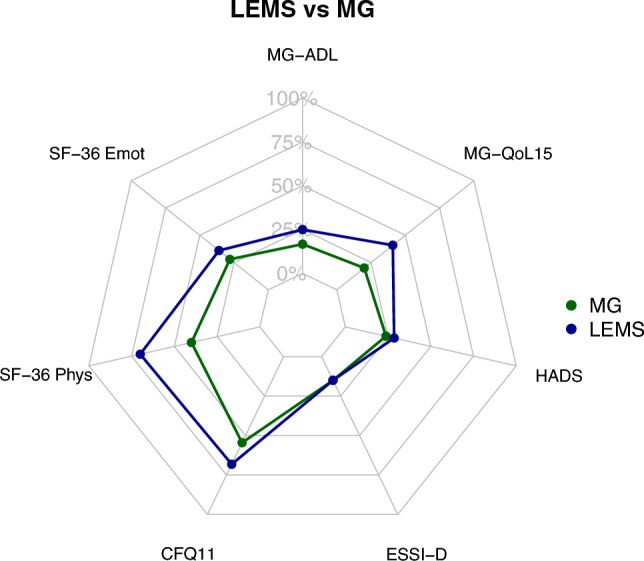
Net diagrams comparing LEMS (blue) and MG (green) patients integrating the medians of the Myasthenia gravis Activities of Daily Living Score (MG-ADL), the Myasthenia gravis Quality of life Score (MG-QoL15), the Hospital Anxiety and Depression Scale (HADS), the ENRICHD Social Support Inventory (ESSI-D), the Chalder Fatigue Scale (CFQ11) and the Physical Functioning (SF-36 Phys) and Emotional wellbeing (SF-36 Emot) domain of the Short Form 36 (SF-36) comparing MG (green) and LEMS (blue) patients. The further out the lines are in the net, the higher and worse the single score value: LEMS patients do have a higher burden of disease, composed of high single score values, compared to MG patients

The MG-ADL and MG-QoL15 were positively correlated (Spearman’s correlation coefficient *r* = 0.60): The more difficulties of daily living have been reported, the lower was the HRQoL measured by MG-QoL15. Longer disease duration was correlated with a lower MG-QoL15 sum score (Spearman’s correlation coefficient *r* = − 0.31). In the HADS anxiety subscale, more than one-third (36.4%) showed 8 points or more, defined as presence of anxiety. In the depression subscale, we found 17.8% of SP with signs of mild depression (8–10 points), 11.1% of SP with severe (11–14 points) and 2.2% of SP with signs of very severe depression (15–21 points). Patients with low social support showed more symptoms of anxiety and depression (median HADS 17, IQR 17;18) and experienced a lower quality of life (median MG-QoL15 29, IQR 19;34) compared to patients with higher levels of social support (median HADS 10, IQR 5.8;15, median MG-QoL15 23, IQR 14.5;28.8); median MG-ADL was only slightly higher (7, IQR 4.5;10.8 vs. 6, IQR 3;9). The individual aspects of the burden of LEMS as captured by the different assessments were summarized in net diagrams (Fig. 3). Both, the SF-36 analysis and the analyses of the aforementioned scores suggest that the overall burden is higher in women and in patients with high disease severity. Compared to MG patients the burden of disease, presented as the interplay of different scores (Fig. 4), is higher in LEMS patients.

## Discussion

In this cross-sectional study with a questionnaire-based survey, we demonstrate that HRQoL is markedly lower in LEMS patients compared to the genP as well as MG. The overall burden is particularly high among women and at high disease severity level. While most patients report alleviation of symptoms under medication, only few experience remission. Correspondingly, symptom burden of LEMS patients as measured by MG-ADL is higher than previously reported in a corresponding cohort of MG patients [16]. LEMS patients additionally report symptoms of autonomic dysfunction.

Few studies have focused on quality of life in LEMS patients [6, 18]. In our in-depth analysis using a matched-pair comparison to the genP in Germany, differences in the domains of the SF-36 indicate a high individual burden for LEMS patients. Because of the similarities between LEMS and MG as disorders of neuromuscular junction transmission, as well as overlap of symptoms and treatment we further compared results from LEMS SP to existing data for MG. Importantly, results from SF-36 analyses indicate that physical limitations are even greater for LEMS SP compared to matched MG patients. In line with our results, the physical composite score in a cohort of 42 Dutch SP with LEMS was significantly lower compared to genP and MG SP. Of note, the majority of the ten questions regarding *physical functioning* of the SF-36 pertain to weakness of the lower extremities. Prominent weakness of legs is typical for LEMS and not as typical in MG [41]. However, beyond exertion-dependent muscular weakness depicted by SF-36 and MG-ADL LEMS SP characteristically show symptoms of autonomic dysfunction which further influences physical well-being for LEMS patients. Furthermore, unlike in MG, LEMS SP reported *pain* with high statistical effect compared to genP. In the cohort reported upon by Lipka et al., a similar association was not found. However, a higher percentage of LEMS SP taking analgesics compared to existing data for MG SP indicates relevance for LEMS patients [16]. Further research into etiology and characteristics of pain in LEMS may elucidate this finding and could possibly enable better treatment and awareness.

In LEMS patients, effects of age on domains such as *physical functioning* and *emotional well-being* were higher than in the genP. Income and education influence HRQoL in LEMS patients. However, with our matched-pair analysis, we demonstrate that there are no major differences of these effects compared to the genP. For *emotional well-being* especially we found only slight differences between LEMS patients and genP as well as MG patients. Our analyses suggest that LEMS patients are affected strongly by limitations of *physical functioning*, but *emotional well-being* is relatively close to genP. Interestingly, while less than 5% of LEMS patients reported *pharmacologic remission*, more than half (67.4%) stated *minimal manifestations* of symptoms and 78.3% of SP were satisfied with medication. Despite having overall worse quality of life even compared to MG patients, LEMS SP appear less affected by the disease emotionally.

In order to investigate potential influencing factors on the individual burden of disease, standardised scores were used, among them the scores of anxiety (HADS-A) and depression (HADS-D), fatigue (CFQ11) and social support (ESSI-D). All SP exceeded the threshold for severe fatigue in the CFQ11. Fatigue has previously been identified as a particularly troublesome symptom in LEMS as well as one of the most limiting symptoms [6, 33]. This may constitute a large impact on overall HRQoL. Anxiety and depression as measured by HADS-D were prevalent in 30–40% of cases. Depression is a common concomitant diagnosis in chronic disease, however, we found no previous reports on incidence in LEMS. For clinical practice a routine screen may be advisable as depression has been shown to increase disease burden substantially and influence quality of life, i.e. for patients with MG or multiple sclerosis and is potentially a treatable complaint [10, 11, 27]. Both the high prevalence of fatigue as well as anxiety and depression stand in contrast with the relatively good emotional well-being derived from SF-36 analysis. Reasons for this discrepancy are unclear.

LEMS SP reported *difficulties getting up from a chair* in most cases (83%) corresponding to proximally pronounced muscle weakness of the legs. Weakness of the legs was named as the most limiting symptom in LEMS by more than half of study participants in another questionnaire-based study [33]. Proximal weakness of the lower extremities as well as *mouth dryness*, which was the most common (80.9%) accompanying symptom in our cohort, have been noted as typical first symptoms in aiLEMS und pLEMS [37]. At time of participation, mean disease duration for our SP was almost 12 years underlining that both symptoms are still highly relevant even in the advanced disease course. Interestingly *fatigue of eyelids* was reported by 73.9% of SP which is higher than previously reported. One study focusing on ocular symptoms in LEMS in 126 patients reported 23% of patients with ptosis [44]. Forms of dyspnea were reported by 68.1% of study participants. We found no previous study focusing on the prevalence of this symptom specifically in LEMS, reports of acute respiratory failure are rare [30]. Concerning accompanying autonomic symptoms mouth dryness (80.9%), as well as dryness of the eyes (74.5%) and obstipation (27.7%) were most common among LEMS SP. Considering that the majority of SP were taking pyridostigmine, 3,4-DAP or 3,4-DAPP with known side effects including hypersalivation, tear fluid increase as well as diarrhea, the high frequency of these complaints is unexpected. Different treatment options for autonomic dysfunction may be necessary. One symptom that may possibly be more common than previously reported in LEMS is ataxia. A recent study found clinical signs of cerebellar ataxia in more than half of SP (56.6%) [24]. Unfortunately, asking patients for signs of ataxia may be prone to errors as diagnosis in clinical practice is generally based on neurological examination. We, therefore, did not include a question concerning ataxia.

Social support was higher in pLEMS SP compared to aiLEMS SP. Possibly, a tumour diagnosis and associated connotations are better known and, therefore, easier to understand for relatives and social support, therefore, higher in pLEMS. Further factors that might have an impact on the perceived burden of disease are partnership and family planning, education level, employment situation and income. 70.2% of LEMS SP were living in a partnership. Compared to MG patients, fewer SP reported an influence of LEMS on family planning and unlike in MG, LEMS as cause of separation was not reported. One reason might be the higher median age of diagnosis in women (LEMS: 51 (IQR 50;59) years vs MG: 45 (30;60) (unpublished data from Lehnerer et al. [16]). At symptom onset, family planning may typically be completed, in our subset, only eight SP had symptom onset before or during family planning. Likewise divorce or separation of study participants possibly occurred before symptom onset (mean age of divorce in Germany 2021 for men 47.0 years, women 43.9 years; mean age of symptom onset LEMS for men 52.8, for women 41.8 [34]). For SP who had been working before the onset of LEMS, limitations regarding employment were common. Almost half of these SP were unable to work. Employment status has been previously reported as an independent factor for worse QoL in LEMS [18].

Concerning limitations, a weakness of our study is that the data for the genP [12] was collected more than 10 years ago and data for comparison to patients with MG [16] was collected about 2 years earlier than LEMS data. Some answers might have changed over time. Self-reporting might impair accuracy concerning medical information. One inherent limitation of questionnaire-based studies is selection bias. SP must be motivated and healthy enough to fill out the questionnaire and able to read and understand the individual items. We, therefore, offered a long response time of four months. Furthermore, the study size is relatively small which is in part due to the rarity of the disease. Overinterpretation must be avoided especially where participant answers were missing and had to be imputed. Gender distribution was weighted; however, more women than would have been expected from epidemiological data were in this cohort [19, 24]. Mean age of onset at 51.9 years was slightly lower than in previous publications that reported age of onset between 55 and 60 years [1, 24, 42]. Importantly, for LEMS, it is possible that patients suffering from an aggressive malignant disease such as SCLC are underrepresented. Median Survival time for LEMS patients with SCLC has been estimated between 17 and 48 months [19, 21, 24]. Furthermore, tumour associated symptoms may lead to less frequent enrolment in a patient organization for LEMS. In our analysis, we included all SP that reported a malignant tumour as pLEMS SP. Reported tumour ethnologies are in line with previous reports from literature [24]. However, it is likely that some of the tumours reported, especially with unclear temporal connection, had no causative connection to LEMS and led to overestimation of pLEMS in our cohort. Additionally, self-reporting led to uncertainties, whenever an exact classification of tumor etiology was not given. For example, the two SP who reported bronchial carcinoma, did not provide any further detail. Our number, even including possible confounders, is still significantly lower than the previously reported number of 50–60% pLEMS which is matching some more recent observations, that pLEMS may be less frequent than previously accepted [22, 41]. However, reports have been varying [18, 28]. Another limitation is that the MG-ADL as well as the MG-QoL15 questionnaire are not validated for LEMS patients and especially for autonomic dysfunction a score specifically designed for LEMS and quantifying all typical symptoms would better elucidate upon the overall picture of the disease. Concerning antibody status more than a quarter of SP reported not knowing. Similarly, high numbers have been published for LEMS patients previously highlighting the necessity of patient education [33]. Notably, three patients reported isolated AChR-Antibodies. In an initial question, patients were asked to state whether they had MG and were included only if this was denied, however, due to the questionnaire-based approach further elucidation was not possible and inaccuracy of antibody-status is possible.

The strengths of our study are the matched-pair analysis, a comprehensive multidimensional approach aiming for inclusion of all-encompassing data, and a representative cohort. Furthermore, the comparison of LEMS not only to genP but also another group of diseased people i.e. MG patients is a strength of this study. To our knowledge, there have been no previous analyses of anxiety and depression in LEMS.

## Conclusion

Our study is a large study on quality of life for LEMS patients in Germany. HRQoL in LEMS patients is remarkably lower in comparison to the genP as well as MG patients. Quality of life reflects one aspect of the burden of disease. Our data demonstrate that many LEMS related as well as unrelated factors contribute like pieces of a puzzle to the burden of disease. For a more disease-specific view of patient´s individual burden of disease development of a LEMS-specific questionnaire encompassing motoric as well as autonomic symptoms that integrates other influencing factors besides quality of life, such as functional level, depression and anxiety, fatigue, and social participation would be desirable. In current phase-III-studies, disease-specific PROMS are the primary and secondary outcome measurements. This highlights that the perceived subjective experience of the individual LEMS patient is the most relevant parameter to improve. Our data warrant the need to conduct prospective multicenter studies to assess the individual burden of disease including generic scores like the SF-36 to make results comparable with the normal population as well as other (neurological) diseases. Special attention should be paid to gender-aspects as women suffering from LEMS do have a higher burden of disease.

### Supplementary Information

Below is the link to the electronic supplementary material.

Supplementary file1 (DOCX 78 kb)

## Data Availability

The study was conducted in accordance to the declaration of Helsinki and the STROBE reporting guidelines.
