# Supplementary material for: Burden of disease in Lambert-Eaton myasthenic syndrome: taking the patient’s perspective
Source: J Neurol. 2024 Feb 29;271(5):2824–39. doi: 10.1007/s00415-024-12206-6 (PMC11055781; doi:10.1007/s00415-024-12206-6)
Supplement: Supplementary file 1 — Supplementary file1 (DOCX 78 kb) [file 415_2024_12206_MOESM1_ESM.docx]

**Supplemental data**

**Burden of disease in Lambert-Eaton myasthenic syndrome – taking the patient’s perspective**

***Journal of Neurology***

Sophie Lehnerer, Meret Herdick^1,2^*, Regina Stegherr, Lea Gerischer, Frauke Stascheit, Maike Stein, Philipp Mergenthaler, Sarah Hoffmann, Andreas Meisel

* *Corresponding author (*[*meret-luise.herdick@charite.de*](mailto:meret-luise.herdick@charite.de)*)*

*1 Charité – Universitätsmedizin Berlin, corporate member of Freie Universität Berlin and Humboldt-Universität zu Berlin, Department of Neurology with Experimental Neurology, Charitéplatz 1, 10117 Berlin, Germany*

*2 Charité – Universitätsmedizin Berlin, corporate member of Freie Universität Berlin and Humboldt-Universität zu Berlin, Department of Neurology with Experimental Neurology, Neuroscience Clinical Research Center, Charitéplatz 1, 10117 Berlin, Germany*

Supplement 1 Number of **missing values** with and without imputation (SF-36 score subscales)

| **Subscales of the SF-36** | Number of missing values | |
| --- | --- | --- |
|  | **without** imputation | **with** imputation |
| Physical functioning | 4 | 0 |
| Physical role functioning | 6 | 4 |
| Vitality | 3 | 2 |
| Social functioning | 8 | 3 |
| Social role functioning | 1 | 1 |
| Emotional well-being | 3 | 2 |
| Pain | 3 | 2 |
| General health perception | 5 | 4 |

Supplement 2 **Additional clinical characteristics** of study participants

| **Age at onset of symptoms** (missing n=2) | |  | **Mean (SD)** |
| --- | --- | --- | --- |
| Total (n=45) | |  | 48.7 (14.0) |
| Men (n=10) | |  | 52.8 (12.9) |
| Women (n=34) | |  | 41.8 (19.6) |
| **Duration from first symptoms to diagnosis (years)** (missing n=3) | | | **Mean (SD)** |
| Total (n=44) | |  | 3.8 (8.2) |
| Men (n=10) | |  | 2.8 (4.1) |
| Women (n=34) | |  | 4.2 (9.0) |
| **Disease duration since diagnosis (years)** (missing n=1) | | | **Mean (SD)** |
| Total (n=46) | |  | 11.8 (7.7) |
| Men (n=9) | |  | 12.7 (9.2) |
| Women (n=36) | |  | 11.6 (7.5) |
| **Comorbid diseases** (multiple answers possible) | |  | **n (%)** |
| Cardiovascular disease | |  | 20 (42.6) |
| Autoimmune disease | |  | 18 (40) |
| Neurologic disease | |  | 13 (27.7) |
| Lung disease | |  | 9 (19.1) |
| Osteoporosis | |  | 8 (17.0) |
| Diabetes mellitus | |  | 6 (12.8) |
| Psychiatric disorder | |  | 2 (4.3) |
| Other diseases | |  | 17 (36.2) |
| No comorbid disease | |  | 14 (29.8) |
| At least 1 comorbid disease | |  | 33 (70.2) |
| At least 2 comorbid diseases | |  | 24 (51.1) |
| 3 and more comorbid diseases | |  | 11 (23.4) |
| **Co-medication** |  |  | **n (%)** |
| Intake of antidepressants (missing n=2) |  |  | 6 (13.3) |
| Intake of painkillers (regularly) (missing n=0) |  |  | 9 (19.1) |

Supplement 3 **Current medication**

| **Current medication** | **missing/**  **not applicable** | **n** | **%** | **% in the last 6 months** | **dosage/d**  **Mean (mg) (STD)** | **missing (dosage)** |
| --- | --- | --- | --- | --- | --- | --- |
| Pyridostigmine | 2 | 18 | 40.0 |  | 146.4 (108.3) | 0 |
| Pyridostigmine sustained release | 2 | 11 | 24.4 |  | - (-) | 11 |
| Glucocorticosteroids | 2 | 11 | 24.4 |  | - (-) | 11 |
| Azathioprine | 2 | 20 | 44.4 |  | 1 (-) | 19 |
| Mycophenolate mofetil | 2 | 0 | 0.0 |  | - (-) | 0 |
| Methotrexate | 2 | 0 | 0.0 |  | - (-) | 0 |
| Cyclosporine A | 2 | 2 | 4.4 |  | 125 (106.1) | 0 |
| Rituximab | 2 | 5 | 11.1 | 60.0 |  |  |
| IVIG | 2 | 21 | 46.7 | 76.2 |  |  |
| Plasmapheresis/Immunoabsorption | 2 | 3 | 6.7 | 0.0 |  |  |
| 3,4-diaminopyridine | 2 | 17 | 37.8 |  | - (-) | 17 |
| Amifampridine | 2 | 22 | 48.9 |  | 49.3 (21.3) | 0 |

Supplement 4 **Matching** LEMS-patients 1:2 with general population (genP, control group, data from DEGS1 study) and 1:2 with MG (Myasthenia gravis) patients (data from MyaBoD study [16]), distribution of gender, age, education and weighted income. Cramer’s V >0.5 indicates a high effect, 0.3-0.5 medium effect, 0.1-0.3 low effect and <0.1 no effect

p=0.002 Cramer’s V=0.191

p<0.001 Cramer’s V=0.275

|  | **Patients with LEMS** | | **Control group** | | **Patients with MG** | |
| --- | --- | --- | --- | --- | --- | --- |
|  | n=46 | | n=92 | | n= 92 | |
| **Gender** | **n** | **%** | **n** | **%** | **n** | **%** |
| Men | 10 | 21.7 | 20 | 21.7 | 20 | 21.7 |
| Women | 36 | 78.3 | 72 | 78.3 | 72 | 78.3 |
| **Age** | **n** | **%** | **n** | **%** |  |  |
| 25 - 29 years old | 1 | 2.2 | 2 | 2.2 | 2 | 2.2 |
| 30 - 39 years old | 2 | 4.3 | 4 | 4.3 | 4 | 4.3 |
| 40 - 49 years old | 2 | 4.3 | 4 | 4.3 | 4 | 4.3 |
| 50 - 59 years old | 13 | 28.3 | 26 | 28.3 | 26 | 28.3 |
| 60 - 69 years old | 9 | 19.6 | 18 | 19.6 | 18 | 19.6 |
| 70 – 74 years old | 5 | 10.9 | 10 | 10.9 | 10 | 10.9 |
| >75 years old | 14 | 30.4 | 28 | 30.4 | 28 | 30.4 |
| **Education (CASMIN)** | **n** | **%** | **n** | **%** | **n** | **%** |
| High | 14 | 30.4 | 13 | 14.6 | 28 | 30.4 |
| Medium | 12 | 26.1 | 33 | 37.1 | 42 | 45.7 |
| Low | 20 | 43.5 | 43 | 48.3 | 22 | 23.9 |
| *Missing* | *0* |  | *3* |  | *0* |  |
| **Income (weighted)** | **n** | **%** | **n** | **%** | **n** | **%** |
| High | 23 | 63.9 | 15 | 16.3 | 36 | 46.1 |
| Medium | 6 | 16.7 | 45 | 34.8 | 18 | 23.1 |
| Low | 7 | 19.4 | 32 | 48.9 | 24 | 30.8 |
| *Missing* | *10* |  | *0* |  | *14* |  |

Supplement 5 **Multivariable analysis on emotional well-being (SF-36)** (combined results after multiple imputation, n=230). (marginal means and 95%CI, model included interaction effect for group*sex and group*age group)

| **Estimated marginal means (95%CI)** | | | | | | |
| --- | --- | --- | --- | --- | --- | --- |
|  | **Controls** | **LEMS patients** | **MG patients** | **Difference (Controls-LEMS)** | **Difference (MG-LEMS)** |  |
| **age group** |  |  |  |  |  | 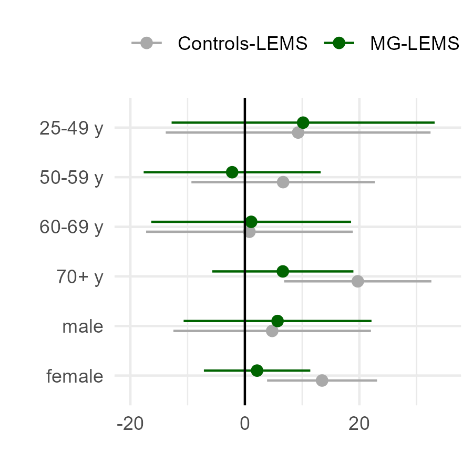 |
| 25-49 y | 75  (64-87) | 66  (50-82) | 76  (65-88) | **9 (-14-32)** | **10 (-13-33)** |  |
| 50-59 y | 75  (67-63) | 68  (57-80) | 66  (58-74) | **7 (-9-23)** | **-2 (-18-13)** |  |
| 60-69 y | 75  (66-85) | 74  (62-86) | 76  (67-85) | **1 (-17-18)** | **1 (-16-19)** |  |
| 70+ y | 80  (74-87) | 61  (52-69) | 67  (61-73) | **20 (7-33)** | **7 (-6-19)** |  |
| **sex** |  |  |  |  |  |  |
| males | 78  (68-87) | 73  (61-85) | 79  (70-87) | **5 (-12-22)** | **6 (-11-22)** |  |
| females | 75  (71-80) | 62  (56-68) | 64  (59-69) | **13 (4-23)** | **2 (7-11)** |  |
| **education** |  |  |  |  |  | **differences between categories**  **(in both groups)** |
| low | 73  (67-79) | 64  (56-72) | 68  (61-74) | **9 (-1-20)** | **4 (-6-14)** | -8 (-18-3) |
| medium | 76  (69-82) | 67  (59-74) | 71  (65-76) |  |  | -5 (-15-5) |
| high | 81  (74-88) | 72  (63-80) | 76  (69-82) |  |  | reference |
| **income** |  |  |  |  |  |  |
| low | 78  (72-84) | 69  (61-77) | 73  (66-79) | **9 (-1-20)** | **4 (-6-14)** | 1 (-9-12) |
| medium | 75  (68-81) | 66  (57-74) | 70  (63-76) |  |  | -2 (-14-10) |
| high | 77  (69-85) | 68  (60-75) | 72  (65-78) |  |  | reference |
| **partnership** |  |  |  |  |  |  |
| no | 77  (69-84) | 68  (59-76) | 72  (65-78) | **9 (-1-20)** | **4 (-6-14)** | 0 (-6-7) |
| yes | 76  (71-81) | 67  (60-75) | 71  (66-76) |  |  | reference |

Supplement 6 Numbers of missing values in Table 4

| **Parameter** | **All** | **Men** | **Women** | **pLEMS** | **aiLEMS** | **high symptom severity** | **low/medium symptom severity** |
| --- | --- | --- | --- | --- | --- | --- | --- |
| n | 47 | 10 | 36 | 10 | 37 | 6 | 41 |
| **Age at diagnosis** (missing)) | 1 | 1 | 0 | 1 | 0 | 0 | 1 |
| **MG-ADL** (missing) | 1 | 0 | 1 | 0 | 1 | 0 | 1 |
| **MG-QoL15** (missing) | 15 | 4 | 11 | 3 | 12 | 1 | 14 |
| **HADS** (missing) | 4 | 0 | 4 | 1 | 3 | 0 | 4 |
| **HADS-A** ≥ 8 p. (missing) | 3 | 0 | 3 | 0 | 3 | 0 | 3 |
| **HADS-D** ≥ 8 p. (missing) | 2 | 0 | 2 | 1 | 1 | 0 | 2 |
| **CFQ** sum (Likert) (missing) | 9 | 2 | 7 | 2 | 7 | 1 | 8 |
| **ESSI-D** ≤ 18 p. (missing) | 3 | 0 | 3 | 0 | 3 | 0 | 3 |
